# Exogenous interleukin-33 promotes hepatocellular carcinoma growth by remodelling the tumour microenvironment

**DOI:** 10.1186/s12967-020-02661-w

**Published:** 2020-12-11

**Authors:** Wenxiu Wang, Jun Wu, Mei Ji, Changping Wu

**Affiliations:** 1 Department of Tumour Biological Treatment, The Third Affiliated Hospital of Soochow University, Changzhou, Jiangsu China; 2 Department of Oncology, The Third Affiliated Hospital of Soochow University, 185 Juqian Street, Changzhou, 213003 Jiangsu China

**Keywords:** Interleukin-33, Hepatocellular carcinoma, Tumourigenesis

## Abstract

**Background:**

Interleukin-33 (IL-33) is an effective inducer of pro-inflammatory cytokines regulating innate and adaptive immunity. Inflammation could be a double-edged sword, promoting or inhibiting tumour growth. To date, the roles and mechanisms of IL-33 in tumours remain controversial. Here, we examined the effect of exogenous IL-33 on the biological characteristics of hepatocellular carcinoma (HCC) and the possible mechanism of action.

**Methods:**

In this study, IL-33 expression in the tissues of 69 HCC patients was detected and its relationship with prognosis was evaluated. After establishing a mouse HCC model and IL-33 treatment operation, the infiltration of splenic myeloid-derived suppressor (MDSCs), dendritic (DCs), regulatory T, and natural killer (NK) cells was detected by flow cytometry analysis, and the vascular density of the tumour tissues was detected by immunohistochemistry to reveal the mechanism of IL-33 in HCC proliferation. Finally, the Cancer Genome Atlas database was used to analyse Gene Ontology terms the and Kyoto Encyclopaedia of Genes and Genomes pathway. Moreover, the chi-square test, two-tailed unpaired Student’s *t*-test, and multiple *t-*tests were performed using SPSS version 23.0 and GraphPad Prism 8.0 software.

**Results:**

The IL-33 expression level was negatively correlated with the overall survival of HCC patients, suggesting its potential clinical significance in the prognosis of HCC. We found that systemic IL-33 administration significantly promoted the tumour size in vivo. Furthermore, the IL-33-treated mice presented decreased frequencies of tumouricidal NK and CD69^+^ CD8^+^ T cells. After IL-33 treatment, the incidence of monocytic MDSCs and conventional DCs increased, while that of granulocytic MDSCs decreased. Moreover, IL-33 promoted the formation of intracellular neovascularization. Therefore, IL-33 accelerated HCC progression by increasing the accumulation of immunosuppressive cells and neovascularization formation. Finally, we found that the transcription of IL-33 was closely related to the PI3K-Akt and MAPK pathways in Gene Set Enrichment Analysis plots, which were involved in the tumourigenesis and pathogenesis of HCC.

**Conclusions:**

Taken together, IL-33 may be a key tumour promoter of HCC proliferation and tumourigenicity, an important mediator, and a potential therapeutic target for regulating HCC progression.

## Background

Interleukin-33 (IL-33) has been described as a potent promoter of type II immunity that triggers an innate immune response to allergic inflammation and parasitic infections [1]. Since 2005 the function of IL-33, as a pleiotropic cytokine, has been widely studied. IL-33 has been found to be involved in the occurrence of many diseases, such as atherosclerosis [2] and obesity [3], and plays a pro-inflammatory role in asthma [4] and antigen-induced arthritis [5]. Endogenous IL-33 is released into the extracellular system, which alerts the immune system after cell damage or necrosis. Activated IL-33 binds to a co-receptor, a heterodimer composed of ST2 and IL1RAP, and initiates inflammatory pathways [6]. In addition, IL-33 recruits signal adapters and kinases to activate transcription factors in tumour cells, which produce the tumour-associated inflammatory microenvironment [7].

ST2 is an IL-33 specific receptor encoded by the gene *IL1RL1*, expressed mainly by immune cells [8]. The combination of IL-33 and ST2 activates a variety of ST2^+^ immune cells including the group 2 innate lymphoid (ILC2), regulatory T (Tregs), and dendritic cells (DCs), thus, inducing the secretion of various chemokines and proinflammatory cytokines and regulating local and systemic immunity [9]. The involvement of IL-33 in innate and adaptive immunity is bidirectional. When exposed to IL-33, DCs could promote the proliferation and differentiation of naive CD4^+^ T cells [10]. IL-33 could also indirectly regulate Tregs by activating ILC2s, DCs, or mast cells [11]. Therefore, IL-33 is the main regulator of Tregs and ILC2s, and has multiple activities in type I, type II, and immune regulation [12].

Further, IL-33 promotes inflammatory events in tumours and activates pro- or anti-tumour responses [13]. In pancreatic cancer, IL-33 is responsible for the upregulation of proinflammatory cytokines, such IL-6 and IL-8 [14]. Transgenic IL-33 activates natural killer (NK) and T cells, resulting in growth inhibition and metastasis of melanoma and lung cancer [15]. The IL-33/ST2 pathway upregulates CD40L and suppresses the growth of murine colon cancer [16]. However, IL-33 enhances type II immune response accelerating tumour progression in tumour-bearing animals [17]. Colon cancer transfection with IL-33 promotes tumour metastasis by accumulating myeloid-derived suppressor cells (MDSCs) to regulate the tumour microenvironment (TME) [18]. ST2^+^ tumour-associated macrophages are recruited into the TME by CXCR3 to suppress host immunity and ST2 knockout mice inhibit colorectal cancer growth in combination with anti-PD-1 antibody [19]. Tumour-derived IL-33 activates mast cells and macrophages, promoting the development of gastric cancer [20]. Therefore, the role of IL-33 in cancer remains controversial. In a previous study on hepatocellular carcinoma (HCC), the RS3821204 genotype of plasma ST2 was positively correlated with HCC risk in Chinese individuals [21]. Interestingly, IL-33 in stromal cells regulated by the pDGF-BB-SOX7 axis promoted HCC metastasis through tumour-associated macrophages [22]. However, tumour-infiltrating effector-memory CD8^+^ T cells in surgically resected tissues producing IL-33 could prolong the survival of HCC patients [23]. In addition, IL-33 released by the liver inhibits HCC growth by promoting T cell response [24]. Therefore, the effect of IL-33 on HCC by the regulation of the immune system should be further studied.

Here, we examined the effect of exogenous IL-33 on the biological characteristics of HCC in human tissues and in mice, and predicted the most relevant pathways of IL-33-associated genes. Our study explored the correlation between IL-33 expression and the prognosis of patients, suggesting that IL-33 may be a marker of poor HCC prognosis. Moreover, the mechanism of IL-33 was explained in terms of tumour microenvironment remodelling, secretion of factors promoting tumour proliferation, and microvascular density. Finally, we proposed the scope for future development direction and research regarding the mechanisms and roles of IL-33.

## Methods

### Samples

The HCC tissue array was purchased from Shanghai Outdo Biotech (Shanghai, China). The cases were enrolled based on their clinical follow-up data and the absence of preoperative history of chemoradiotherapy. The Edmondson and Steiner classification was used to grade the tumour [25]. The TNM staging was used in accordance with the American Joint Committee on Cancer guidelines. The age of the 69 HCC patients in the HCC tissue array ranged from 16 to 75 years (mean age, 49 years). The follow-up time ranged from 0.36 to 155 months (mean time, 32.8 months). Sixty-nine para-cancer tissues pathologically diagnosed as normal were obtained from tissues ≥ 5 cm away from the tumour of HCC patients. Five para-cancer tissue samples were damaged during the heat-induced antigen retrieval process. Finally, a total of 69 HCC patients and 64 paired para-carcinoma controls were included in the analysis. Information on the relationship between IL-33 and clinicopathological features of the HCC patients is presented in Tables 1 and 2. The study protocol was approved by the ethics committee of the Third Affiliated Hospital of Soochow University and conducted in accordance with the Declaration of Helsinki. All patients provided informed consent for participation.

**Table 1:** Correlation between IL-33 expression and clinicopathological characteristics of HCC

Clinicopathological characteristics	Cases	IL-33 expression level		χ^2^	P value
		Low (H-score < 68)	High (H-score ≥ 68)		
Age (years)					
60	55	28	27	0.289	0.591
≥ 60	14	6	8		
Sex					
Male	59	28	31	0.538	0.463
Female	10	6	4		
Number of tumours					
Single	40	18	22	0.696	0.404
Multiple	29	16	13		
Tumour size (cm)					
≤ 5	18	11	7	1.365	0.243
> 5	51	23	28		
Capsule integrity					
No	39	20	19	0.145	0.704
Yes	30	14	16		
HBV positivity					
Negative	8	1	7	4.896	*0.027*
Positive	61	33	28		
TNM stage					
I + II	30	13	17	0.75	0.387
III + IV	39	21	18		
Edmondson type					
I + II	45	24	21	0.852	0.356
III	24	10	14		
Cirrhosis background					
Absent	11	3	8	2.535	0.111
Present	58	31	27		
Venous invasion					
No	61	32	29	2.133	0.144
Yes	8	2	6		
Recurrence					
No	46	24	22	0.464	0.496
Yes	23	10	13		
Metastasis					
No	65	33	32	1.001	0.317
Yes	4	1	3		

Italic font indicates significant differences (P < 0.05)

**Table 2:** Cox-regression analysis of the correlation between IL-33 expression and prognostic parameters in HCC patients

Parameter	Univariate analysis		Multivariate analysis	
	HR (95% CI)	P value	HR (95% CI)	P value
Age (year)				
(≥ 60 vs. < 60)	0.873 (0.452–1.684)	0.685	1.681 (0.797–3.543)	0.172
Number of tumours				
(Multiplies vs. Single)	*1.579 *(*0.937–2.659*)	*0.086*	*1.863 *(*1.053–3.297*)	*0.033*
Sex				
(Female vs. male)	*0.341 *(*0.135–0.861*)	*0.023*	*0.351 *(*0.131–0.939*)	*0.037*
Tumour size (cm)				
(> 5 vs. ≤ 5)	1.576 (0.862–2.883)	0.14	1.333 (0.680–2.616)	0.403
Capsule integrity				
(Yes vs. no)	*0.513 *(*0.300–0.879*)	*0.015*	0.637 (0.358–1.131)	0.124
Venous invasion				
(Yes vs. no)	*2.54 *(*1.217–5.302*)	*0.013*	*2.596 *(*1.140–5.910*)	*0.023*
IL-33 expression				
(High vs. low)	*1.890 *(*1.111–3.216*)	*0.019*	*1.906 *(*1.099–3.305*)	*0.022*

Italic font indicates significant differences (P < 0.05)

*HR* hazard ratio, *CI* confidence interval

### Cell culture

Hepa1-6, an HCC cell line in mice, was purchased from the National Platform for Experimental Cell Resources (Beijing, China). Hepa1-6 cells were maintained in DMEM (Hyclone Laboratories, Logan, UT, USA), and 10% (v/v) foetal bovine serum (ExCell Bio, Clearwater, FL, USA) was added. Cell lines were routinely examined for cell morphology and mycoplasma.

### Experimental animals

Several female C57BL/6 mice (age, 6–8 weeks) were obtained from Shanghai Laboratory Animal Centre (Shanghai, China). These mice were placed in specific pathogen-free facilities. All animal treatment procedures, such as mouse care and experimental procedures, were reviewed by the Animal Ethics Committee of The Third Affiliated Hospital of Soochow University. The animals were fed in strict accordance with operating procedures and provided a clean and comfortable living environment; the animals were handled at appropriate intervals, and measures were taken to minimise animal suffering.

### HCC model

A murine HCC model was established in the current study. A suspension of Hepa1-6 cells was prepared using phosphate-buffered saline (PBS), before implantation. Then, the syngeneic female mice (age, 6–8 weeks) were anesthetised with chloral hydrate. A total of 8 × 10^6^ Hepa1-6 cells resuspended into 80 µL PBS were injected subcutaneously on day 0 into the back of the mice. Then, the mice were injected with the murine recombinant IL-33 protein (0.4 µg/mouse; PeproTech, Cranbury, NJ, USA) or PBS intraperitoneally five times every other day starting from day 3. Finally, the mice were sacrificed, and tumour volumes were calculated using the following formula:

$$ {\text{Tumour volume}} = \left( {{\text{length}} \times {\text{width}}^{{2}} } \right)/{2}. $$

### Immunohistochemistry

Tissue sections were incubated with primary antibodies for anti-human IL-33 (R&D Systems, Minneapolis, MN, USA), anti-mouse Ki67, CD31, SA1009, vascular endothelial growth factor (VEGF; all obtained from Abcam, Cambridge, UK), or matching IgG isotypes overnight. Then, the sections were conjugated with secondary antibody. For the tissue array, Pannoramic MIDI (3DHISTECH, Budapest, Hungary) was used to scan the tissue points. All the immunohistochemical reactions were evaluated by three experienced pathologists who were blinded to clinical information according to the H-score method [26]. The H-score was calculated as follows:

$$\begin{aligned}   {\text{H-score}} &  = \left( {{\text{percentage of cells of weak intensity}}} \times {1} \right) + \left( {{\text{percentage of cells of moderate intensity}} \times 2} \right) \\     & \quad  + \left( {{\text{percentage of cells of stringe intensity}} \times 3} \right) \\  \end{aligned}$$

The maximum H-score was defined as 300, based on 100% cells exhibiting strong intensity. The experiment was independently repeated three times.

### Cell proliferation assay

The cell proliferation potential was performed with the cell counting kit-8 assay (CCK8; Invitrogen, Waltham, MA, USA) in vitro. Hepa1-6 cells (5000 cells per well plate) were plated to 96-well plates with IL-33 concentrations of 10, 20, 50, and 100 ng/ml for 48 h and, then, detected by CCK-8 (Dojindo Laboratories, Kumamoto, Japan) with a microplate reader. The experiment was independently repeated three times.

### Quantitative reverse-transcription polymerase chain reaction (qPCR)

RNA from murine HCC was extracted with Trizol reagent (Invitrogen). Especially, 1 μg RNA was reverse transcribed into cDNA with RevertAid First Strand cDNA Synthesis Kit (Servicebio, Wuhan, China) and, then, qPCR was performed using FastStart Universal SYBR Green Master (Servicebio) with the Prism 7900H system (Thermo Fisher Scientific, Waltham, MA, USA). We calculated the relative quantification of mRNA expression using the delta-delta Ct method [27] and normalised the PCRs utilising *GAPDH* as the reference gene. The primers are listed in Additional file 1: Table S1. The experiment was independently repeated three times.

### Flow cytometric analyses

Single cell suspensions of splenocytes and fresh tumour tissues from mice were incubated with fluorochrome-labelled anti-mouse antibodies specific for CD45, CD3, CD4, CD8, NK1.1, Ly6G, CD69, CD44, CD62L, CD11b, CD11c, Gr1, Ly6C, major histocompatibility complex (MHC) class II, B220, or isotype-matched controls (eBioscience, Thermo Fisher Scientific). For the foxp3 nuclear protein, cells were permeabilised using Transcription Factor Staining Buffer Set (Invitrogen) for 30 min. Then, they were analysed using FACSCalibur Flow Cytometer (BD Pharmingen, San Jose, CA, USA). The experiment was independently repeated three times.

### Statistical analysis

The chi-square, two-tailed unpaired Student’s *t*-, and multiple *t-*tests were performed using SPSS (version 23.0; IBM Corp., Armonk, NY, USA) and GraphPad Prism 8.0 (San Diego, CA, USA) software. Overall survival (OS) and disease-free survival (DFS) curves were conducted with the log-rank test according to the Kaplan–Meier method. Data (means ± standard deviations) were repeated at least three independent times. The statistical significance level was set at P < 0.05. Gene Ontology (GO), Kyoto Encyclopaedia of Genes and Genomes (KEGG), and Gene Set Enrichment Analysis (GSEA) were performed with R package clusterProfiler, R package DOSE, and R package org. Hs.eg.db (R Foundation for Statistical Computing, Vienna, Austria). For GSEA plots, false discovery rate q-value (P. adjust) ≤ 0.25, nominal P-value < 0.01, and |normalised enrichment score|≥ 1 were regarded as significant.

## Results

### IL-33 is upregulated in HCC

**Fig. 1:**
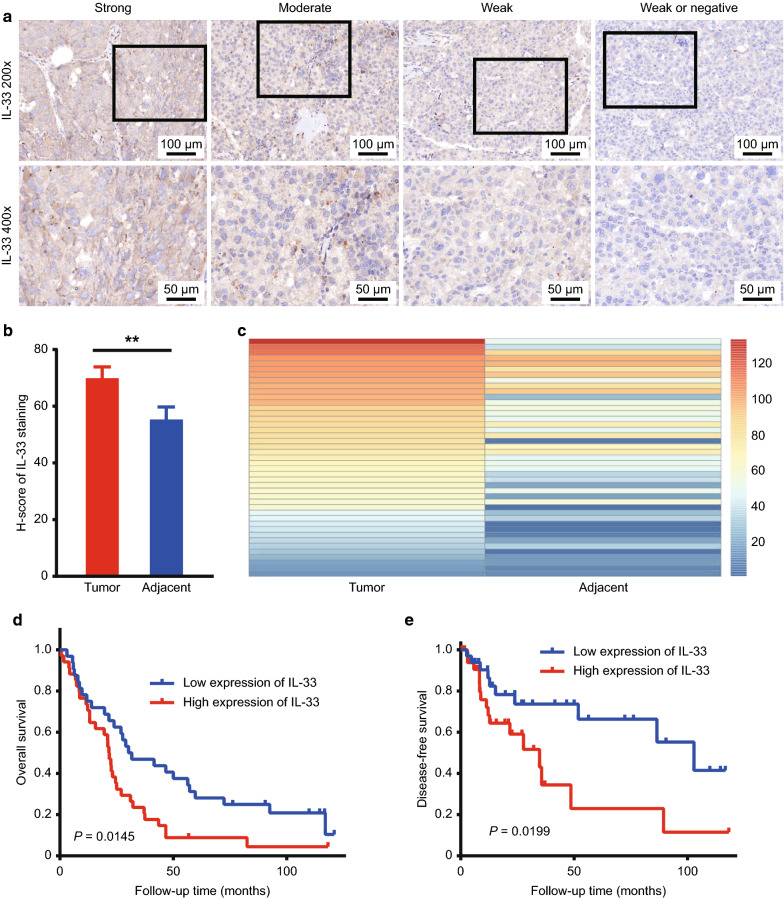
IL-33 expression was upregulated in human HCC tissues and was associated with poor patient survival. **a** The representative IHC images of IL-33 expression in HCC (left-strong, moderate, weak) and in adjacent normal tissues (right-weak or negative) were presented. **b** IL-33 expression was frequently upregulated in 69 HCC tissues compared with that in the 64 adjacent para-carcinoma tissues. **c** After excluding the influence of individual differences, the expression of IL-33 was analysed in 64 paired HCC and adjacent para-cancer tissues in this dataset. **d** Kaplan–Meier OS and **e** DFS curves for 69 HCC patients were analysed based on the H-score of IL-33 staining. *HCC* hepatocellular carcinoma, *IL-33* interleukin-33, *OS* overall survival, *DFS* disease-free survival

To determine the role of IL-33 in HCC patients, we initially explored IL-33 expression in a tissue array containing 69 HCC and 64 paired adjacent normal tissue samples (five para-cancer tissue samples were damaged during the heat-induced antigen retrieval process). Immunostaining with different intensities of IL-33 was shown in the cytoplasm in HCC tissues (Fig. 1a; Left-strong, moderate, weak). Weak or negative staining (Fig. 1a; right) of IL-33 was found in the adjacent normal tissues. Through the quantitative analysis of IL-33 expression, we found that it was upregulated in the HCC tissue compared with that in the adjacent tissue (Fig. 1b). To exclude the influence of individual differences, we further analysed IL-33 expression in 64 paired HCC and adjacent para-cancer tissues in this dataset and found that it was significantly upregulated in the 44 paired HCC tissues compared with the adjacent tissues (Fig. 1c).

### High IL-33 expression in HCC correlates with poor patient survival and advanced clinicopathological features

To understand the clinical implications of upregulating IL-33, we analysed the correlation between IL-33 expression and clinicopathological characteristics in HCC patients. Higher IL-33 expression was significantly correlated with Hepatitis B virus positivity (χ^2^ = 4.896; P = 0.027) but not with sex, tumour size, TNM stage, or differentiation (Table 1).

We further analysed the clinical follow-up time of 69 HCC patients. Compared with patients with low IL-33 expression, those with high IL-33 expression had significantly shorter OS (P = 0.0145; hazard ratio [HR], 1.853; 95% confidence interval [CI] 1.096–3.134) (Fig. 1d). Moreover, the differences were even more significant in the DFS of HCC patients (P = 0.0145; HR, 2.428; 95% CI 1.107–5.326) (Fig. 1e). We also analysed the effect of IL-33 expression on the prognosis of HCC. In univariate Cox regression analyses, high expression of IL-33 was associated with increased death risk in HCC patients (P = 0.019; Table 2). Furthermore, IL-33 expression in HCC patients (P = 0.022) could be identified as an independent prognostic predictor in multivariate Cox regression analysis (Table 2).

### Exogenous IL-33 leads to accelerated HCC growth

**Fig. 2:**
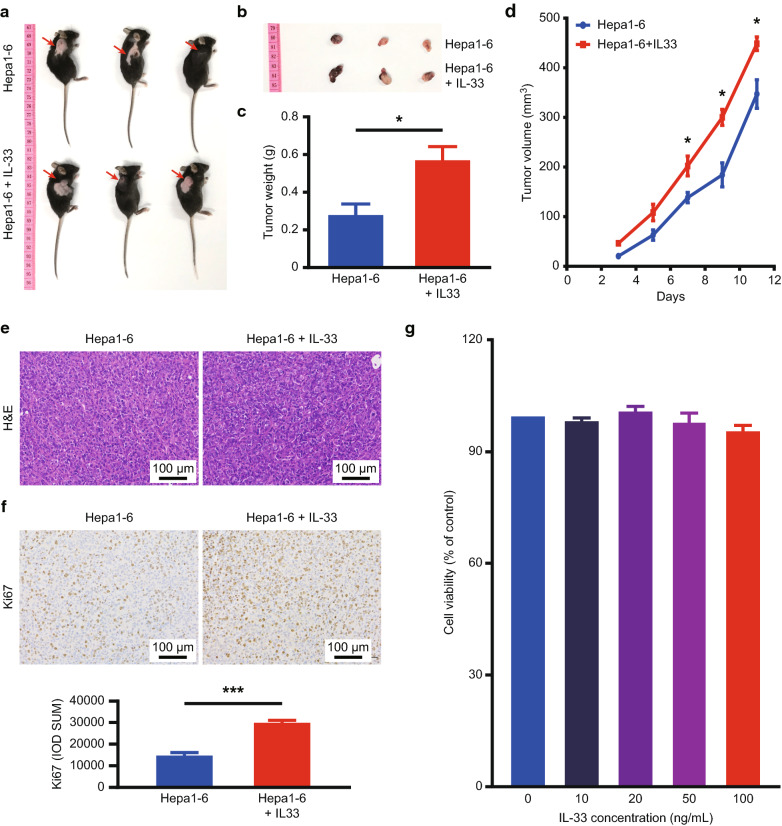
IL-33 enhanced HCC tumourigenesis. C57BL/6 mice were injected subcutaneously with Hepa1-6 cells (8 × 10^6^) and treated with or without IL-33 protein (0.4 µg/mouse) in PBS intraperitoneally every other day. **a** Gross view of mice and **b** tumours after euthanasia of mice. **c** An histogram of tumour weight was presented from both groups of mice. **d** Tumour volume changed over time in two groups. **e** H&E staining images of tumour tissues from both groups of mice were presented. **f** Ki67 protein expression was detected by IHC. **g** The effect of IL-33 on Hepa1-6 cell proliferation was observed by CCK8 in vitro. *PBS* phosphate-buffered saline, *H&E* haematoxylin and eosin, *IHC* immunohistochemistry, *CCK8* cell counting kit 8

To examine the effect of IL-33 on tumour progression, the murine HCC model was established in Hepa1-6 cells. HCC developed aggressively in the IL-33-treated experimental group, as reflected by the gross view of the mice (Fig. 2a) and tumour (Fig. 2b, Additional file 2: Figure S1a**)**. The tumour weight was significantly higher in the IL-33-treated than in the PBS-treated mice (Fig. 2c, Additional file 2: Figure S1b). In addition, the tumour volume increased rapidly after IL-33 treatment, with significant difference at multiple time points compared with that in untreated mice (Fig. 2d, Additional file 3: Figure S2c). Therefore, IL-33 treatment was found to dramatically promote Hepa1-6 growth in vivo*.*

Although no significant difference was observed in haematoxylin and eosin (H&E) staining between mice treated with IL-33 or those that were not, it was clear that Hepa1-6 cells were loosely arranged with large nuclear malformation and imbalance of nuclear plasma in these mice (Fig. 2e, Additional file 2: Figure S1d). The staining intensity of the proliferative marker Ki67 in the IL-33-treated experimental group was stronger and more significant compared with that in the control group (Fig. 2f, Additional file 2: Figure S1e). This suggested that IL-33 augmented the proliferation of Hepa1-6 HCC tissues in mice. However, IL-33 had no direct effect on the proliferation of Hepa1-6 cells in vitro (Fig. 2g). The efficacy of IL-33 may be partly attributable to tumour immunogenicity rather than to the changing intrinsic characteristics of tumour cells, as reported in literature [28].

### IL-33 may remodel the tumour microenvironment (TME)

**Fig. 3:**
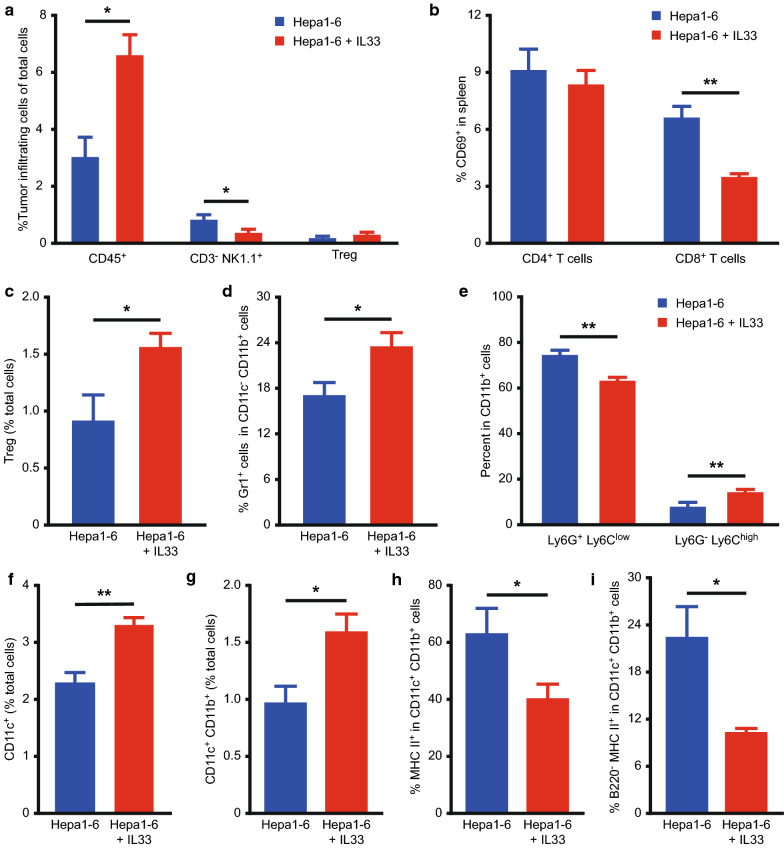
IL-33 inhibited immune effector cells and mobilised immunosuppressive cells into the TME. Splenocytes and intrahepatic single-cell suspensions were harvested for flow cytometric analysis. **a** Percentages of tumour-infiltrating CD45^+^ leukocyte, CD3^−^NK1.1^+^ NK cells, CD4^+^Foxp3^+^ Tregs among total cells were determined by flow cytometry. **b** The percentages of activated CD69^+^CD4^+^ T and CD69^+^CD8^+^ T cells were presented. **c** IL-33 induced accumulation of splenic CD4^+^Foxp3^+^ Tregs. **D**, **e** Flow cytometry was used to analyse Gr1, Ly6G, and Ly6C expression in gated CD11c^−^CD11b^+^ cells. **d** IL-33 treatment increased the accumulation of CD11c^−^CD11b^+^Gr1^+^ MDSCs in tumour-bearing mice. **e** The percentages of CD11c^−^CD11b^+^Ly6G^+^Ly6C^low^ granulocytic and CD11c^−^CD11b^+^Ly6G^−^Ly6C^high^ monocytic MDSCs were presented before and after IL-33 treatment. **f–i** Flow cytometry was used to analyse CD11c^+^, CD11c^+^CD11b^+^ in spleens, and MHCII^+^ and B220^−^MHCII^+^ expression in gated CD11c^+^CD11b^+^ cells. IL-33 increased the percentage of CD11c^+^ (**f**) and CD11c^+^CD11b^+^ cells (**g**), while it decreased the percentage of conventional CD11c^+^CD11b^+^B220^−^MHCII^+^ DCs cells (**h**, **i**). *TME* tumour microenvironment, *MDSCs* myeloid-derived suppressor cells, *DCs* dendritic cells, *IL* interleukin, *Tregs* regulatory T cells

To investigate whether exogenous IL-33 affects HCC occurrence by regulating the immune system, we examined immune cell phenotypes in the spleen and tumours of Hepa1-6-bearing mice treated with IL-33 and those who were not (Fig. 3). IL-33 administration markedly increased CD45^+^ leukocyte immune cell infiltration on day 11 after the tumour challenge (Fig. 3a). Moreover, we observed a decrease in frequencies of intratumoural NK cells in tumour-bearing mice with IL-33 treatment (Fig. 3a). The percentage of activated CD69^+^CD8^+^ T cells was remarkably downregulated in the IL-33-treated group (Fig. 3b). However, effector (CD44^+^CD62L^−^) or naive (CD44^−^CD62L^+^) CD4^+^ T and CD8^+^ T cells were not affected by IL-33 administration (Additional file 3: Figure S2a, b). In addition, no significant difference was observed in the mRNA levels of IFN-γ between the two groups (Additional file 3: Figure S2c). These results demonstrated that IL-33 may promote tumour progression by reducing the proportion of CD8^+^ T and NK cells in vivo.

Meanwhile, IL-33 increased the splenic infiltration of immunosuppressive cells in tumour-bearing mice. Tregs, which play critical roles in immunosuppression, were significantly increased in spleens when compared with the corresponding in PBS-treated mice (Fig. 3c), but not in the tumour tissue (Fig. 3a). Moreover, IL-33-treated mice exhibited a pronounced increase in CD11c^−^CD11b^+^Gr1^+^ MDSCs, which led to tumour immune escape and tumourigenesis (Fig. 3d). Monocytic MDSCs increased and granulocytic MDSCs decreased after IL-33 treatment in tumour-bearing mice compared with that in the control group, albeit not significantly (Fig. 3e). Furthermore, IL-33 administration influenced the incidence of splenic DC subpopulations. Significant increases in the percentage of CD11c^+^ and CD11c^+^CD11B^+^ DCs were found in tumour-bearing mice treated with IL-33 (Fig. 3f, g).

Moreover, the MHC II expressed on DCs had an effect on evoking efficient anti-tumour immune responses. MHC II expression in splenic DCs decreased in response to IL-33 administration, thus, blocking DC maturation and their cross-presentation ability (Fig. 3h). In addition, IL-33 treatment reduced the percentage of CD11c^+^CD11b^+^B220^−^MHCII^+^ conventional DCs (Fig. 3i).

### IL-33 induces tumour-promoting cytokine and neomicrovessel formation

**Fig. 4:**
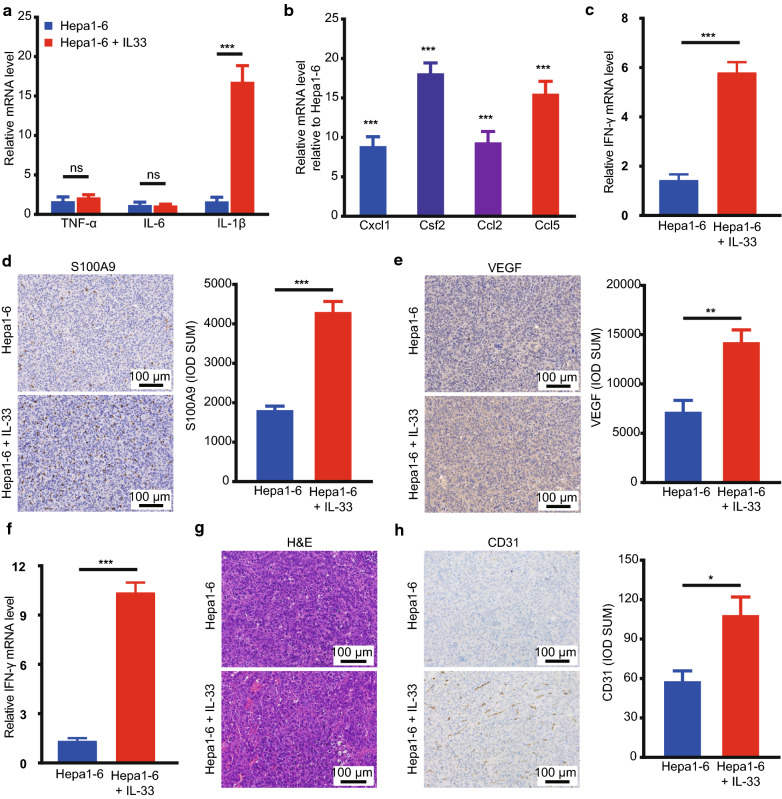
IL-33 induced tumour-promoting cytokine and neomicrovessel formation. **a** The mRNA levels of tumour-promoting cytokines IL-6, IL-1β, and TNF-α in tumour tissues from both groups of mice were presented. **b** Quantitative mRNA analysis of chemokines Csf2, Ccl2, Ccl5, and Cxcl1 in tumours of IL-33-treated mice compared with those in PBS-treated wild-type mice. **c** Relative S100A9 mRNA levels and its immunohistochemical staining (**d**) were presented before and after IL-33 treatment. **e–f** VEGF was detected at the protein (**e**) and mRNA levels (**f**) in two groups. **g**, **h** Tumour sections from both groups of mice were stained with H&E (**g**) and IHC staining of CD31 (**h**) to detect microvessel formation. *IHC* immunohistochemistry, *PBS* phosphate-buffered saline, *IL* interleukin, *VEGF* Vascular endothelial growth factor, *TNF* tumour necrosis factor

To investigate the influence of exogenous IL-33 on pro-inflammatory factors, such as IL-6, IL-1β, and tumour necrosis factor (TNF)-α, we measured their mRNA expressions, which are known to stimulate tumour proliferation and angiogenesis [29]. Although there was a slight difference in the IL-6 and TNF-α mRNA levels, the IL-1β cytokine levels were elevated in tumours injected with IL-33 (Fig. 4a). Then, we detected the chemokines that recruited myeloid cells into the TME and mediated angiogenesis [30]. The results showed that the mRNA levels of Csf2, Ccl2, Ccl5, and Cxcl1 chemokines significantly increased after IL-33 treatment (Fig. 4b). In addition, exogenous IL-33 enhanced the immunostaining intensity and mRNA levels of S100A9 (Fig. 4c, d), which induced epithelial-mesenchymal transition and led to tumour occurrence [31]. Therefore, IL-33 may induce chemokines (e.g. Cxcl1), which enhanced the recruitment of myeloid cells secreting S100A9 to promote tumour progression.

Moreover, the microvascular density was approximately higher in experimental than in untreated mice (Fig. 4g), indicating significant microvascular generation. A past study showed that IL-33 could initiate vascular remodelling through upregulating VEGF expression in hypoxic pulmonary hypertension [32]. As a key signal to stimulate angiogenesis, VEGF was highly expressed after IL-33 treatment, supporting our previous conclusion (Fig. 4e, f). In addition, IL-33 elevated the immunohistochemistry (IHC) staining intensity of endothelial cell marker like CD31, an indicator of neomicrovessels (i.e. blood and lymphatic vessels), which suggested that active angiogenesis and lymphangiogenesis occurred in IL-33-treated tumours (Fig. 4h).

### Predictions of the functions and pathways of IL-33 and the genes significantly associated with IL-33 alterations

**Fig. 5:**
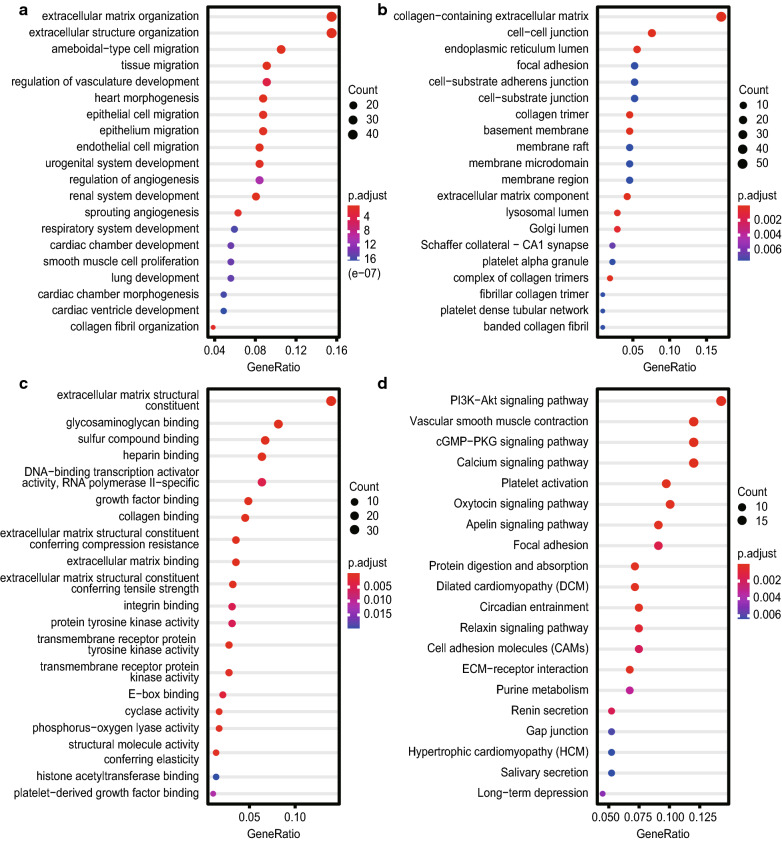
Predicted functions and pathways of IL-33 and genes were significantly associated with IL-33 alterations. **a**–**d** Tοp 300 genes with the highest positive correlation genes were selected for enrichment analysis of GO terms (**a**–**c**) and KEGG pathway (**d**). GO analysis predicted three main functions including biological processes (**a**), cellular components (**b**), and molecular functions (**c**). *GO* Gene Ontology, *KEGG* Kyoto Encyclopedia of Genes and Genomes, *IL-33* interleukin-33

To explore the functions of IL-33 and the genes that were significantly associated with IL-33 alterations, GO and KEGG pathway enrichment analyses were performed. As presented in Fig. 5a–c, biological processes, such as GO: 0030198 (extracellular matrix organization), GO: 0043062 (extracellular structure organization), GO: 0043542 (endothelial cell migration), GO: 0001667 (ameboid-type cell migration), and GO: 0090130 (tissue migration), were remarkably regulated by the IL-33 alterations in HCC patients (Fig. 5a). Cellular components and molecular functions including GO: 0062023 (collagen-containing extracellular matrix), GO: 0005911 (cell–cell junction), GO: 0005201 (extracellular matrix structural constituent), and GO: 0005539 (glycosaminoglycan binding) were significantly associated with these IL-33 alterations (Fig. 5b, c). The KEGG pathway analysis showed that the most enriched pathways were 04151 (PI3K-Akt signalling pathway), 04022 (cGMP-PKG signalling pathway), 04020 (Calcium signalling pathway), 04611 (Platelet activation), and 04371 (Apelin signalling pathway), which were associated with tumourigenesis and HCC progression (Fig. 5d).

**Fig. 6:**
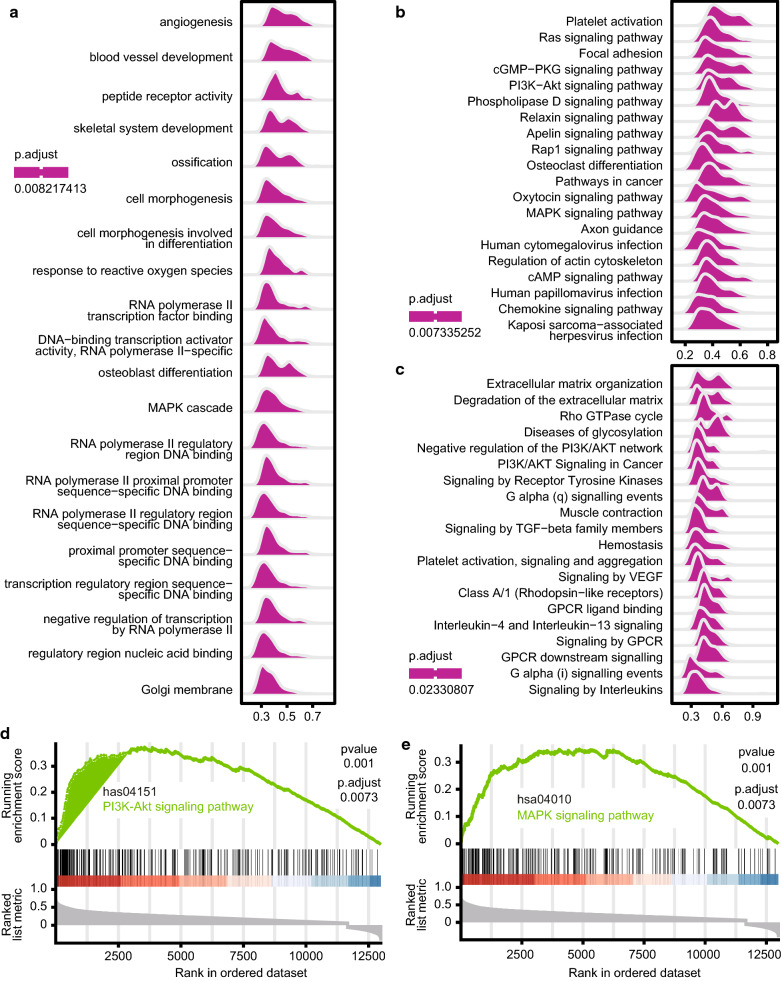
GSEA using TCGA database. **a** Ridge plot of GSEA-based GO terms, **b** KEGG pathways, and **c** Reactome analyses were performed using the HCC TCGA database. **d**–**e** Correlations between IL-33 transcripts, PI3K-Akt pathway (**d**), and MAPK pathway (**e**) were performed in GSEA-based KEGG-enrichment plots. The green line indicated the enrichment profile. *TCGA* the cancer genome atlas, *KEGG* Kyoto Encyclopedia of Genes and Genomes, *GSEA* Gene set enrichment analysis, *HCC* hepatocellular carcinoma, *GO* Gene Ontology, *IL-33* interleukin-33

In addition, GSEA was conducted to search GO terms, KEGG pathways, and Reactome using the HCC TCGA database. In these ridge plots, many pathways involved in tumourigenesis and cell cycle were related to IL-33 alterations (Fig. 6a–c). Among these pathways, we listed the two most common functional gene sets enriched in HCC with high IL-33 expression. GSEA-based KEGG-enrichment plots showed that the gene signatures of the PI3K-Akt (Fig. 6d) and MAPK signalling pathways (Fig. 6e) were highly correlated with IL-33 expression, which contributed to the tumourigenesis and progression of HCC.

## Discussion

Our study examined the effects of exogenous IL-33 on the biological characteristics of HCC in human tissues and in mice. We observed a higher IL-33 expression in liver cancer than in para-cancer tissues (Fig. 1a–c). Meanwhile, we verified that increased IL-33 expression was closely related to short OS and DFS in HCC patients (Fig. 1d, e). Studies have shown that the serum IL-33 [33] and ST2 [34] levels were associated with a worsened clinical prognosis of various diseases. Our study suggested that IL-33 may be a marker of poor prognosis in HCC patients. Repeated injection of IL-33 accelerated liver cancer growth in mice, as shown by the increased tumour size, weight, and Ki67 expression in the HCC tissue sections (Fig. 2a–f). However, IL-33 did not demonstrate Hepa1-6 cell proliferation in vitro, excluding its direct effect on tumour cells (Fig. 2g). Therefore, we hypothesized that IL-33 could play a role in tumour occurrence by influencing the TME [12], similar to its role in breast cancer [17].

Exogenous IL-33 increased CD45^+^ leukocyte immune cells and decreased NK cell infiltration in tumours (Fig. 3a). The percentage of activated CD69^+^CD8^+^ T cells in the spleen was significantly downregulated in IL-33-treated mice (Fig. 3b). This reflects the important pathophysiological role of exogenous IL-33 in immune effector cells. Contrary to our findings, several studies have shown that IL-33 promotes type I immune response. Indeed, the antiviral immune response requires IL-33 signalling in CD8^+^ T cells [35]. IL-33 enhances NK and CD8^+^ T cell function, thereby, inhibiting tumour growth in transgenic mice [36]. High levels of IL-33 promote invasion of the TME by CD8^+^ T cells, NK, and NKT where they mediate their antitumour response [37]. The underlying cause of the difference between our findings and the corresponding of these studies remains unclear. We speculated that the immune effect of IL-33 may be influenced by its dose and microenvironment.

Several studies have supported the pro-tumour effect of IL-33, in line with our findings. Low levels or systemic IL-33 administration might induce immune tolerance in mice [38]. IL-33 administration promotes stemness in colon cancer cells by recruiting macrophages [39]. This may be because systemic injection of IL-33 elicits a broad immune response that promotes tumour growth in mice. MDSCs, Tregs, and immature DCS were treated as inhibiting antitumour immunity and promoting tumour angiogenesis [40]. IL-33 promoted the accumulation of MDSCs in the spleen of mice (Fig. 3d) and affected the frequency of monocytic and granulocytic MDSCs (Fig. 3e). However, the immunosuppressive ability of different subsets is not clear [41]. MDSCs are recruited from peripheral lymphoid organs to tumour sites, promoting the production of CD4^+^Foxp3^+^Tregs [42]. Our results showed increased DCs (Fig. 3f, g), whereas CD11c^+^CD11b^+^B220^−^MHCII^+^ conventional DCs decreased in response to IL-33 (Fig. 3i). Therefore, we speculated that IL-33 might increase plasmacytoid DCs in the TME, showing a powerful immunosuppressive property by inhibiting T cell activation or promoting Tregs development [43], which is consistent with the increase in Tregs frequency in our data (Fig. 3c). In addition, IL-33 induces immature DCs, which have poor stimulation and lower MHC II expression (Fig. 3h). IL-33 accelerated HCC progression by increasing the accumulation of immunosuppressive cells and inhibiting immune effector cells.

IL-33 enhanced the mRNA levels of pro-inflammatory factor TNFα, which might stimulate tumour proliferation and angiogenesis (Fig. 4a). Further exploration revealed that chemokines (e.g. Cxcl1), which might favour the recruitment of myeloid cells into the TME, were significantly increased after IL-33 treatment (Fig. 4b). Once myeloid cells were recruited into the TME, pro-inflammatory cytokines that promote tumour proliferation were secreted [44]. Thus, increased S100A9 was found at the RNA and protein levels after IL-33 treatment (Fig. 4c, d), which may provide a pre-metastatic niche that could lead to tumour occurrence. In addition, IL-33 induced angiogenesis and vascular permeability, as demonstrated in a study of a murine asthma surrogate [45]. In our IHC staining data, CD31 and VEGF expression was significantly increased in HCC tissues after treatment with IL-33 (Fig. 4e, h). This was consistent with the higher VEGF levels, as observed in the PCR test (Fig. 4f), and the higher microvascular density (Fig. 4g) observed in the experimental group.

IL-33 is considered to be a reactive inducer of the immune system to inflammation and malignancy. Studies have shown that the presence of some inflammatory factors could also affect the biological function of IL-33. IL-2 and IL-33 synergistically induce IFN-γ and enhance IL-33-driven immune response. High levels of IL-33 in the presence of pathogen-associated molecular pattern–induced IL-12 promote type I anti-tumour immune response [46]. In the presence of immunosuppressive factors (e.g. tumour growth factor-β), tumour stromal IL-33 plays an immunosuppressive role through Tregs and MDSCs.

IL-33, as an effective vaccine adjuvant combined with human papilloma virus vaccine enhances anti-tumour immunity of CD8^+^ T cells in vivo [47]. In addition, the combination of IL-33 blockers with programmed death-1 monoclonal antibodies could successfully inhibit acute myeloid leukaemia, and its combination with imatinib could also prevent chronic myeloid leukaemia resistance [48]. The effect of antibody drugs against IL-33 could be examined through clinical trials for asthma and other diseases. IL-33 mediates the invasion of tumour-associated fibroblasts promoting the development of head and neck squamous carcinoma in the TME [49], suggesting that IL-33 may be a key mediator between the stromal cells and tumour. However, the manner in which IL-33 expression regulates tumour epithelial and stromal cells remains unclear. Considering the dual role of IL-33 in cancer, it might be considered carefully in drug development as a target. In particular, IL-33 has the potential to cause inflammation and cancer.

## Conclusions

In this study, we examined the effect of exogenous IL-33 on the biological characteristics of hepatocellular carcinoma in humans and in mice. We found that the overall survival of patients with high IL-33 expression was significantly shortened, suggesting that IL-33 may be a marker of poor prognosis in HCC patients. IL-33 favoured lymphocyte invasion of the tumour microenvironment, thus, promoting tumour growth and angiogenesis. The mechanism of IL-33 was explained in terms of tumour microenvironment remodelling, secretion of factors promoting tumour proliferation, and microvascular density. IL-33 may be a key tumour promoter that promotes the proliferation and tumourigenicity of HCC. Considering that IL-33 could play a dual role in cancer, it might be considered carefully in drug development as a target.

## Supplementary Information

**Additional file 1: Table S1.** The primers used in the study.

**Additional file 2: Figure S1.** Exogenous IL-33 leaded to accelerate HCC growth.

**Additional file 3: Figure S2.** IL-33 did not affect effector or naive T cells and mRNA levels of IFN-γ.

## Data Availability

The data that support the findings of this study are available from the corresponding author upon reasonable request.

## Abbreviations

IL-33: Interleukin-33
HCC: Hepatocellular carcinoma
NK: Natural killer
DCs: Dendritic cells
cDCs: Conventional dendritic cells
OS: Overall survival time
DFS: Disease-free survival
MDSCs: Myeloid-derived suppressor cells
GO: Gene Ontology
KEGG: Kyoto Encyclopedia of Genes and Genomes
ILC2: Group 2 innate lymphoid cells
Tregs: Regulatory T cells
TME: Tumour microenvironment
MHC: Major histocompatibility complex
IHC: Immunohistochemistry
