# Supplementary material for: Exogenous interleukin-33 promotes hepatocellular carcinoma growth by remodelling the tumour microenvironment
Source: J Transl Med. 2020 Dec 11;18:477. doi: 10.1186/s12967-020-02661-w (PMC7733302; doi:10.1186/s12967-020-02661-w)
Supplement: Supplementary file 1 — Additional file 1: Table S1. The primers used in the study. [file 12967_2020_2661_MOESM1_ESM.docx]

**Table S1** The primers used in the study.

| **Gene** | **Forward primer (5’-3’)** | **Reverse primer (5’-3’)** |
| --- | --- | --- |
| IFN-γ | CTCAAGTGGCATAGATGTGGAAG | TGACCTCAAACTTGGCAATACTC |
| Csf2 | AAAGAAGCCCTGAACCTCCTG | GAAATCCGCATAGGTGGTAACTT |
| Cxcl1 | CACCCAAACCGAAGTCATAGC | GGGGACACCTTTTAGCATCTTT |
| IL-6 | CCCCAATTTCCAATGCTCTCC | CGCACTAGGTTTGCCGAGTA |
| IL-1β | TCAAATCTCGCAGCAGCACATC | CGTCACACACCAGCAGGTTATC |
| TNF-a | ACCCTCACACTCACAAACCA | ATAGCAAATCGGCTGACGGT |
| Ccl2 | ACCAGCAAGATGATCCCAATG | GTGCTTGAGGTGGTTGTGGA |
| Ccl5 | CGCACCTGCCTCACCATAT | TGTAGAAATACTCCTTGACGTGGG |
| GAPDH | CCTCGTCCCGTAGACAAAATG | TGAGGTCAATGAAGGGGTCGT |
