# Supplementary material for: Exogenous interleukin-33 promotes hepatocellular carcinoma growth by remodelling the tumour microenvironment
Source: J Transl Med. 2020 Dec 11;18:477. doi: 10.1186/s12967-020-02661-w (PMC7733302; doi:10.1186/s12967-020-02661-w)
Supplement: Supplementary file 2 — Additional file 2: Figure S1. Exogenous IL-33 leaded to accelerate HCC growth. [file 12967_2020_2661_MOESM2_ESM.docx]

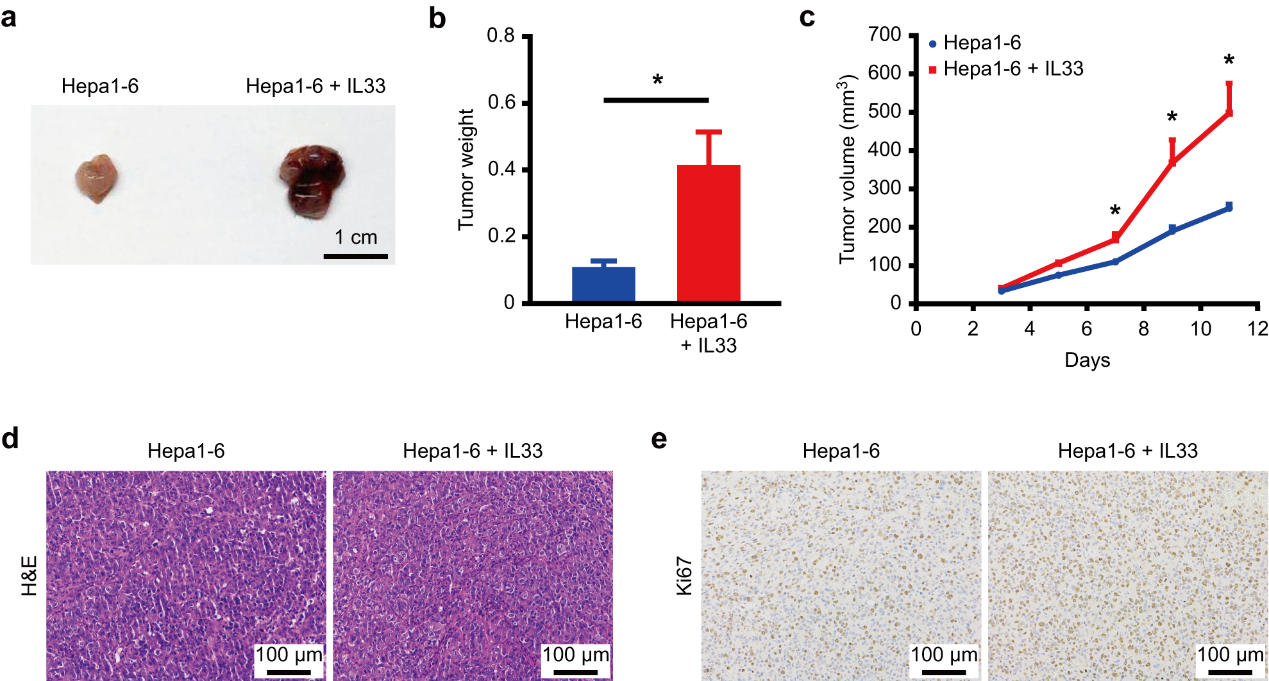


**Fig S1.** Exogenous IL-33 leads to accelerate HCC growth. C57BL/6 mice were subcutaneously injected with Hepa1-6 cells (5×10^6^) and treated with intraperitoneal recombinant IL-33 (0.4 µg/mouse) in PBS or PBS alone every other day. **a** Gross view of tumors from both groups of mice after euthanasia. **b** [Histogram](javascript:;) of tumor weight is shown. **c** Tumor volume changed over time in two groups. **d** H&E staining images of tumor tissues are shown. **e** HCC tumor sections stained with anti Ki67 antibodies.

PBS, phosphate-buffered saline; HCC, hepatocellular carcinoma
