# Supplementary material for: Exogenous interleukin-33 promotes hepatocellular carcinoma growth by remodelling the tumour microenvironment
Source: J Transl Med. 2020 Dec 11;18:477. doi: 10.1186/s12967-020-02661-w (PMC7733302; doi:10.1186/s12967-020-02661-w)
Supplement: Supplementary file 3 — Additional file 3: Figure S2. IL-33 did not affect effector or naive T cells and mRNA levels of IFN-γ. [file 12967_2020_2661_MOESM3_ESM.docx]

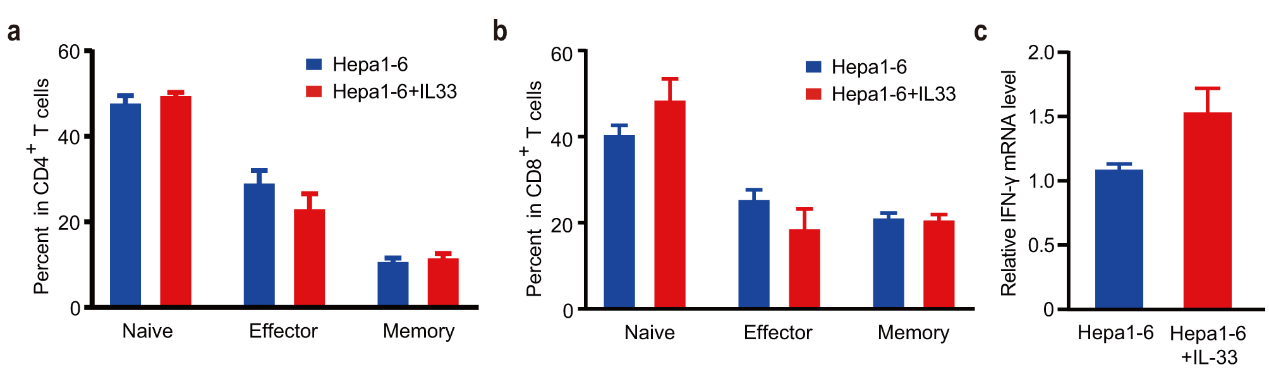


**Fig S2.** IL-33 did not affect effector or naive T cells and mRNA levels of IFN-γ. Percentage of effector (CD44^+^CD62L^-^), naïve (CD44^-^CD62L^+^), and memory (CD44^+^CD62L^+^) in CD4^+^ T cells (**a**) and CD8^+^ T cells (**b**) in spleens are shown. **c** Quantitative RNA analysis of IFN-γ in two groups.
